# Ocular Surface Recovery Following Corrective Eyelid Surgery: Course, Predictors, and Perioperative Management Implications

**DOI:** 10.3390/diagnostics16182977

**Published:** 2026-09-15

**Authors:** Maria Angela Romeo, Alberto Cuccu, Valerio Calabresi, Filippo Lixi, Giulia Coco, Vanessa Ferraro, Alessandro Gaeta, Silvia Ferraro, Giuseppe Giannaccare, Alessandra Di Maria

**Affiliations:** 1 Department of Ophthalmology, University Magna Graecia of Catanzaro, 88100 Catanzaro, Italy; 2 Eye Clinic, Department of Surgical Sciences, University of Cagliari, 09124 Cagliari, Italyvalerio.calabresi1@gmail.com (V.C.);; 3 Ophthalmology Unit, Department of Clinical Sciences and Translational Medicine, University of Rome Tor Vergata, 00133 Rome, Italy; 4 Ophthalmology Unit, AUSL-IRCCS di Reggio Emilia, Viale Risorgimento 80, 42123 Reggio Emilia, Italy; vanessaferraro94@gmail.com; 5 Department of Internal Medicine and Medical Specialties (DIMI), Università di Genova, 16132 Genova, Italy; 6 Department of Ophthalmology, University of Catania, 95124 Catania, Italy; silviaferraro97@gmail.com; 7 Department of Ophthalmology, IRCCS Humanitas Research Hospital, 20089 Milan, Italy; alessandra.di_maria@humanitas.it

**Keywords:** dry eye disease, eyelid malposition, ptosis, ectropion, ocular surface, OSDI, tear film, perioperative management, risk stratification

## Abstract

**Background/Objectives:** The aim of this study was to evaluate ocular surface recovery following reconstructive eyelid surgery, to identify preoperative predictors of incomplete symptomatic recovery, and to propose a risk stratification framework to enable targeted perioperative management. **Methods**: This retrospective multicenter study included 50 eyes of 32 patients (mean age 73.0 ± 13.9 years; 44% female) affected by ptosis (eyes = 23) or ectropion (eyes = 27) who underwent standardized corrective surgery at two Italian centers. Ocular surface evaluation was performed preoperatively (T0) and 1 week (T1), 1 month (T2), and 3 months (T3) postoperatively. Outcomes included the Ocular Surface Disease Index (OSDI) questionnaire, Schirmer I test, non-invasive tear breakup time (NI-BUT), and corneal fluorescein staining (Oxford staining score). Pre-existing dry eye disease (preDED) was defined as OSDI ≥ 23 combined with at least one objective sign using conservative thresholds (NI-BUT < 5 s, Schirmer I ≤ 7 mm, or Oxford staining score ≥ 2). **Results**: At 3 months, patient-level OSDI decreased by a median of 11.0 points (*p* < 0.001) and eye-level Oxford staining score by 0.96 grades (*p* < 0.001, r = 0.870). Ectropion patients achieved greater OSDI improvement than ptosis patients (median 21 vs. 2 points, *p* = 0.002) yet converged to comparable final values (median 16 vs. 18, *p* = 0.715). PreDED was identified in 10 patients (32%), all with severe baseline disease (OSDI ≥ 33 plus objective signs). All preDED patients (100%) achieved the minimal clinically important difference (≥7.3 points) versus 33% of no-DED patients (*p* < 0.001). However, 50% of preDED patients maintained moderate symptoms (OSDI ≥ 23) at 3 months versus 19% of no-DED patients (*p* = 0.105). Overall, the assessed parameters suggested a biphasic postoperative course, with transient worsening at 1 week, followed by progressive recovery by 3 months. **Conclusions:** Reconstructive eyelid surgery produces sustained ocular surface improvement within 3 months, yet preDED was associated with incomplete symptomatic recovery despite universal achievement of clinically meaningful improvement. These exploratory findings suggest that patients with severe baseline preDED may benefit from preoperative identification and individualized counseling regarding realistic expectations; whether intensified perioperative management improves outcomes should be tested prospectively. Randomized trials are needed to determine whether preoperative optimization with anti-inflammatory therapy and meibomian gland treatment improves outcomes in this higher-risk subgroup.

## 1. Introduction

The ocular surface depends on precise coordination between eyelid anatomy, blink dynamics, and tear film mechanics. Eyelid malpositions disrupt this equilibrium through multiple mechanisms: altered lid–globe congruity compromises the tribological interface between the lid wiper epithelium and the corneal surface, impaired blink dynamics reduce shear-driven tear redistribution, and loss of anatomical apposition accelerates evaporative loss [1]. These mechanical disturbances destabilize the tear film, a complex non-Newtonian fluid whose rheological properties depend on mucin concentration and lipid layer integrity [2,3,4,5]. When eyelid malposition persists, chronic friction and exposure trigger inflammatory cascades that perpetuate dry eye disease even after the mechanical insult is removed. Similar patterns of ocular surface disruption, including exposure keratopathy, conjunctival scarring, and eyelid malposition, have been documented following periocular surgical interventions, topical pharmacological treatments, and neurological conditions affecting eyelid function, underscoring the vulnerability of the ocular surface to anatomical and functional disruption [6,7,8,9,10].

Ectropion and ptosis represent distinct pathophysiological entities. Ectropion causes loss of lid–globe apposition with direct evaporative exposure and paradoxical epiphora, while ptosis impairs superior blink excursion and tear clearance without overt exposure [11]. Both conditions predispose to dry eye disease (DED), a multifactorial symptomatic disorder affecting 5 to 50% of adults and characterized by loss of tear film homeostasis [12]. The lacrimal functional unit integrates the lacrimal glands, ocular surface epithelia, meibomian glands, eyelids, and sensory-motor neural pathways to maintain this homeostasis; disruption of any component initiates inflammatory amplification and epithelial damage [13].

Reconstructive eyelid surgery aims to restore anatomical alignment and functional protection, yet the course of ocular surface recovery following correction remains incompletely characterized. Existing studies have examined the ocular surface impact of cosmetic upper blepharoplasty and isolated ptosis repair [5,6,7,8,9,10,11,12,13,14,15,16], but few have prospectively compared different malposition types using validated multiparametric dry eye assessment. To our knowledge, no study has specifically compared ocular surface outcomes between ectropion repair and ptosis correction despite their divergent pathophysiology: lateral tarsal strip procedure directly restores lid–globe apposition and reduces corneal exposure, while levator advancement primarily affects upper lid position and blink dynamics [17].

The role of pre-existing DED as a modifier of surgical outcomes remains insufficiently characterized. Recent evidence suggests that pre-existing DED influences postoperative outcomes after levator advancement [18], yet the magnitude of symptomatic improvement in this subgroup has never been quantified using minimal clinically important difference thresholds. The minimal clinically important difference (MCID) for the Ocular Surface Disease Index (OSDI), validated at 7.3 to 13.4 points depending on baseline severity [19], provides an objective benchmark for clinically meaningful improvement but has not been applied in reconstructive eyelid surgery. This gap is clinically significant: patients with severe baseline disease may require different perioperative counseling and management strategies, yet current practice lacks evidence-based risk stratification.

Moreover, the distinction between statistical improvement and complete symptomatic resolution remains unexplored. Patients may achieve clinically meaningful improvement by MCID criteria yet retain moderate symptoms requiring ongoing management. The prevalence and predictors of such incomplete recovery have not been systematically evaluated. Current management also lacks standardization, with variable approaches to anti-inflammatory therapy, lubrication, and meibomian gland optimization across institutions.

This study addresses these gaps by evaluating ocular surface recovery following ptosis correction and ectropion repair in patients with and without pre-existing severe DED. We employed multiparametric assessment including validated symptom questionnaires, tear film stability measurements, tear production testing, and epithelial integrity grading at standardized intervals. Our objectives were to characterize the longitudinal course of ocular surface recovery, to explore whether pre-existing severe DED was associated with incomplete symptomatic recovery despite anatomical correction, and to compare outcomes between ectropion and ptosis patients using MCID thresholds.

## 2. Materials and Methods

Study design and setting: This retrospective observational multicenter study reviewed medical records of patients who underwent corrective eyelid surgery between October 2023 and September 2025 at two Italian centers: the Eye Clinic, Department of Surgical Sciences, University of Cagliari (Center A) and the Department of Ophthalmology, IRCCS Humanitas Research Hospital, Rozzano, Milan (Center B). All patients had been evaluated according to a standardized clinical protocol including preoperative assessment (T0) and postoperative follow-up at 1 week (T1), 1 month (T2), and 3 months (T3). This study was conducted in accordance with the Declaration of Helsinki; approval for retrospective data analysis was obtained from the Ethics Committee of IRCCS Humanitas Research Hospital (protocol code 4566, approval date: 23 September 2025). The requirement for informed consent was waived given the retrospective nature of this study and the use of de-identified data.

Study population: Adults diagnosed with involutional eyelid ptosis or ectropion scheduled for corrective surgery were eligible. Exclusion criteria were previous ocular or eyelid surgery; congenital, neurogenic, mechanical, or traumatic ptosis; systemic diseases known to affect the ocular surface (thyroid eye disease, autoimmune disease, Sjögren syndrome); pre-existing corneal disease; current or prior use of vitamin A (retinoid) derivatives; and current contact lens use. A total of 50 eyes from 32 patients were included (Table 1). Because OSDI is a patient-reported outcome, symptom-based analyses were interpreted with caution in patients contributing both eyes. Eye-level analyses were retained for objective ocular surface parameters, while sensitivity analyses using one eye per patient and GEE models were performed to account for within-patient correlation. Bilateral eyes were included as separate observations given their distinct clinical status; sensitivity analyses restricted to one eye per patient confirmed consistent direction and magnitude of all primary findings. A sensitivity analysis restricted to one eye per patient (worst-eye selection based on highest baseline Oxford staining score, with ties broken by highest OSDI then lowest NI-BUT) was performed to confirm the robustness of primary findings (*n* = 32 patients).

Surgical techniques: All surgeries were performed by a single experienced oculoplastic surgeon in each center using standardized techniques. For ptosis correction, levator aponeurosis advancement was performed in cases of aponeurotic ptosis with levator function ≥ 10 mm (*n* = 18 eyes); frontalis suspension using autologous fascia lata was performed in cases with poor levator function < 5 mm (*n* = 5 eyes) [17]. For ectropion repair, the lateral tarsal strip (LTS) procedure was performed for involutional ectropion with horizontal lid laxity (*n* = 27 eyes) [17].

Postoperative management protocol: All patients at both centers received standardized perioperative management consisting of topical antibiotic-corticosteroid ointment (tobramycin 0.3%/dexamethasone 0.1%) applied to the periocular area 4 times daily for 10 days, and preservative-free artificial tears instilled in the conjunctival sac 4 times daily for the duration of follow-up [20,21,22]. No additional anti-inflammatory therapy or meibomian gland treatment was prescribed. Postoperative complications were systematically documented at each follow-up visit. Given the recognized association between lower eyelid surgery, lateral canthal procedures, and persistent conjunctival chemosis, postoperative chemosis was systematically recorded at each follow-up visit [6]. Other acute complications, including hematoma, infection, wound dehiscence, and lagophthalmos, were similarly recorded.

Outcome measures: Patients were evaluated preoperatively (T0) and 1 week (T1), 1 month (T2), and 3 months (T3) postoperatively. Primary outcomes were as follows:

(i.) Subjective symptoms: Ocular Surface Disease Index (OSDI, 0–100) [19];
(ii.) Tear film stability: NI-BUT measured by Keratograph 5M (Oculus, Wetzlar, Germany) at Center A and MS-39 anterior segment OCT (CSO, Ferrara, Italy) at Center B, both previously validated for tear film evaluation in perioperative settings; NI-BUT was analyzed separately by center due to known inter-device variability (Section 2) [23,24,25];
(iii.) Tear production: the Schirmer I test without topical anesthesia [26];
(iv.) Epithelial integrity: the Oxford corneal and conjunctival staining score (0–5) [12].

Clinically meaningful improvement was defined as an OSDI reduction ≥ 7.3 points (MCID) [19]. Given that all preDED patients had severe baseline DED (OSDI ≥ 33), the MCID range for severe disease is 7.3–13.4 points. Both thresholds were applied: the lower bound (7.3) as the primary, more permissive criterion, and the upper bound (13.4) as a more stringent sensitivity criterion, so that MCID achievement is reported across the full severity-adjusted range rather than relying on a single cutoff [19].

Definition of pre-existing DED: PreDED was defined at baseline as OSDI ≥ 23 (moderate-to-severe symptoms per validated OSDI severity classification) combined with at least one objective sign using conservative thresholds: NI-BUT < 5 s, Schirmer I ≤ 7 mm, or Oxford staining score ≥ 2. While the TFOS DEWS III diagnostic methodology identifies NI-BUT < 10 s as abnormal [12,19,27], these thresholds were deliberately set as stringent, study-specific criteria to isolate a subgroup with objectively confirmed, clinically significant tear film instability and aqueous deficiency, rather than to replicate any single consensus diagnostic cutoff. These conservative criteria were designed to identify patients with objectively confirmed, clinically significant DED requiring targeted perioperative management. All 10 preDED patients (100%) had baseline OSDI ≥ 33, placing the entire subgroup in the severe DED category [19]; thus, findings specific to this subgroup apply to severe DED and may not generalize to moderate DED (OSDI 23–32).

Statistical analysis: Normality was assessed using Shapiro–Wilk tests. All continuous outcomes violated normality (all *p* < 0.05); therefore, non-parametric tests were used throughout. Longitudinal changes were analyzed using Friedman tests with Wilcoxon signed-rank post hoc comparisons versus baseline and Bonferroni correction (α = 0.017 for three comparisons per outcome). Between-group comparisons used Mann–Whitney U tests. Categorical outcomes were analyzed using Fisher’s exact test. Effect sizes are reported as matched-pairs rank-biserial correlation r for Wilcoxon signed-rank tests and rank-biserial correlation r for Mann–Whitney U tests (small 0.1–0.3; medium 0.3–0.5; large > 0.5). Statistical significance was set at *p* < 0.05 (two-tailed). NI-BUT was analyzed separately by center due to device-specific measurement characteristics (Keratograph 5M vs. MS-39 OCT). To account for intra-patient correlation arising from bilateral eye inclusion and repeated measurements, Generalized Estimating Equations (GEEs) were used as a sensitivity analysis for Oxford staining score and Schirmer I. The GEE model used a Gaussian family with identity link, exchangeable working correlation structure, and robust sandwich variance estimation, with patient as the clustering unit (32 clusters, 50 eyes). Fixed effects included timepoint, diagnosis group (ptosis vs. ectropion), and their interaction, with preoperative ptosis as the reference category. NI-BUT was excluded from GEE analysis given the known non-interchangeability of absolute values between the two devices used at the two centers. A sensitivity analysis restricted to one eye per patient (worst-eye selection based on highest baseline Oxford staining score, with ties resolved by highest OSDI then lowest NI-BUT; *n* = 32 patients) was also performed. Because OSDI is a patient-reported outcome, symptom-based eye-level findings were interpreted in conjunction with clustered GEE and one-eye-per-patient sensitivity analyses. All analyses were conducted in Python 3 (SciPy v1.11, NumPy v1.24).

## 3. Results

Study population: Fifty eyes from 32 patients (mean age 73.0 ± 13.9 years, range 26–92; 44% female) were included: 23 eyes with eyelid ptosis and 27 eyes with ectropion (Table 1). Fifteen eyes were evaluated at Center A (Keratograph 5M; 6 ptosis and 9 ectropion) and 35 at Center B (MS-39; 17 ptosis and 18 ectropion). Both malposition types were represented at each center in similar proportions (ectropion 60% at Center A vs. 51% at Center B), indicating that diagnosis was not confounded with center or measuring device. Because a single surgeon operated at each center, however, surgeon, center, and NI-BUT device were mutually nested and could not be disentangled; NI-BUT was therefore analyzed separately by center and never pooled. At the patient level, preDED was identified in 10 patients (32%): 2/12 ptosis patients and 8/19 ectropion patients (eye-level: 18/50 eyes, 36%; 4/23 ptosis and 14/27 ectropion, *p* = 0.018). All preDED patients had baseline OSDI ≥ 33 (100% severe DED category). Among postoperative complications, chemosis was documented in six eyes from three patients (12% of eyes) at the 1-week visit. All cases resolved spontaneously by 1 month without requiring intervention. No cases of hematoma, infection, wound dehiscence, or persistent lagophthalmos were observed.

Baseline characteristics: Numerically higher patient-level OSDI was observed (median 37.0 vs. 20.5), although this difference was not significant (*p* = 0.109), and lower NI-BUT at Center B (5.59 ± 1.66 vs. 9.96 ± 0.98 s, *p* < 0.001). Baseline Schirmer I was comparable between groups (10.6 ± 4.6 vs. 12.0 ± 2.8 mm, *p* = 0.066) (Table 1).

**Figure 1:**
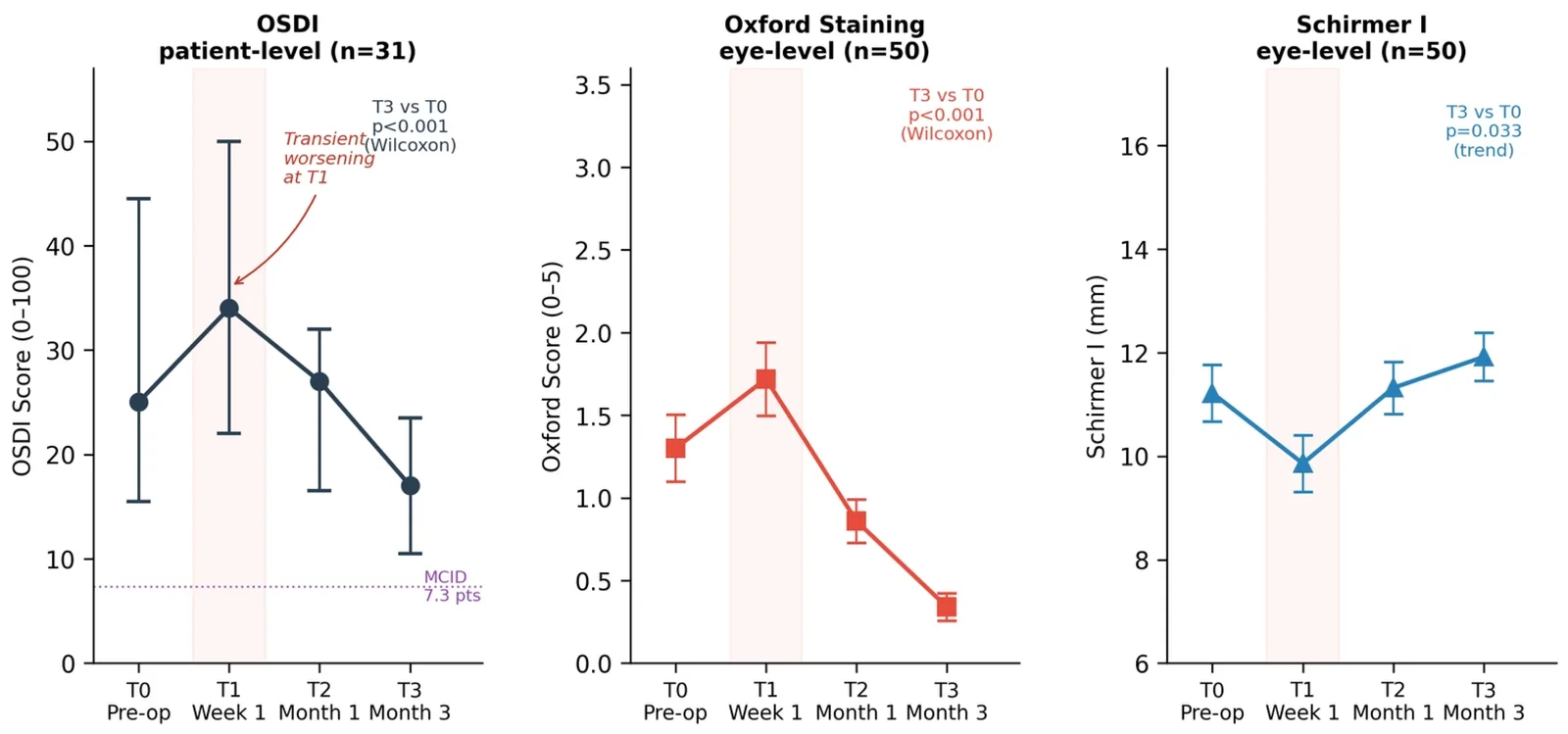
Biphasic ocular surface recovery following corrective eyelid surgery. OSDI is shown at the patient level (*n* = 31 patients; median [IQR]); Oxford staining and Schirmer I are shown at the eye level (*n* = 50 eyes; mean ± SEM). Timepoints: T0, pre-operative baseline; T1, 1 week; T2, 1 month; T3, 3 months. Pink shading indicates the acute postoperative phase (Week 1). All three parameters showed transient worsening at T1 followed by progressive improvement below baseline at T3. The dotted line in the OSDI panel indicates the minimal clinically important difference (MCID) threshold (7.3 points). All within-group comparisons versus baseline: Wilcoxon signed-rank test with Bonferroni correction (α = 0.017). The Schirmer I improvement at T3 (*p* = 0.033) did not pass the Bonferroni-corrected threshold and represents a positive trend only. OSDI, Ocular Surface Disease Index; IQR, interquartile range; SEM, standard error of the mean; MCID, minimal clinically important difference.

Overall longitudinal changes: All three pooled outcome measures demonstrated significant overall change across timepoints (Friedman test, all *p* < 0.001; Table 2). (i.) Patient-level OSDI (*n* = 31) increased from a median of 25.0 [IQR 15.5–44.5] at T0 to 34.0 [22.0–50.0] at T1, then decreased to 27.0 [16.5–32.0] at T2 and 17.0 [10.5–23.5] at T3 (median change −11.0 points from baseline, *p* < 0.001); (ii.) Oxford staining increased at T1 (+0.42 grades, *p* < 0.001, r = 0.870) before declining to 0.34 ± 0.59 at T3 (Δ = −0.96 grades, *p* < 0.001, r = 0.870). (iii.) Schirmer I decreased at T1 (−1.4 mm, *p* < 0.001) and showed a positive trend at T3 (+0.7 mm, *p* = 0.033, r = 0.541), which did not pass the Bonferroni-corrected threshold (α = 0.017) (Figure 1).

NI-BUT by center: At Center A (Keratograph 5M, *n* = 15), overall change was significant (Friedman χ^2^ = 13.0, *p* = 0.005), though no individual timepoint comparison reached significance after Bonferroni correction. At Center B (MS-39, *n* = 35), NI-BUT showed significant overall change (Friedman χ^2^ = 79.3, *p* < 0.001): a decline at T1 (Δ = −1.91 s, *p* < 0.001, r = 0.872) followed by an improvement at T3 (Δ = +1.88 s above baseline, *p* < 0.001, r = 0.761). In the ectropion subgroup at Center B, NI-BUT increased from 5.59 s at T0 to 9.04 s at T3 (Δ = +3.45 s, *p* < 0.001); ptosis showed no significant change (Δ = +0.22 s, *p* = 0.255). Baseline NI-BUT differed between devices in the ptosis subgroup (Center B: 9.96 ± 0.98 vs. Center A: 3.26 ± 1.04 s, *p* = 0.0004); ectropion baseline values were comparable (*p* = 1.000).

Ptosis versus ectropion: At the patient level, 12 patients were included in the ptosis group and 19 patients in the ectropion group. Baseline OSDI was numerically higher in ectropion patients (median 37.0 [22.0–47.5]) than in ptosis patients (20.5 [13.75–25.75]), although the difference was not statistically significant (*p* = 0.109). Both groups showed significant within-group improvement at 3 months (ptosis: median 2.0 [1.0–3.25] points, *p* = 0.005; ectropion: 21.0 [10.0–23.5] points, *p* < 0.001). Improvement was significantly greater after ectropion repair (*p* = 0.002; |r| = 0.658), yet 3-month OSDI values were comparable between groups (18.0 [14.25–21.5] vs. 16.0 [6.0–26.5], *p* = 0.715). The ≥7.3-point MCID was achieved by 2/12 ptosis patients (16.7%) and 15/19 ectropion patients (78.9%; Fisher exact *p* = 0.001).

**Figure 2:**
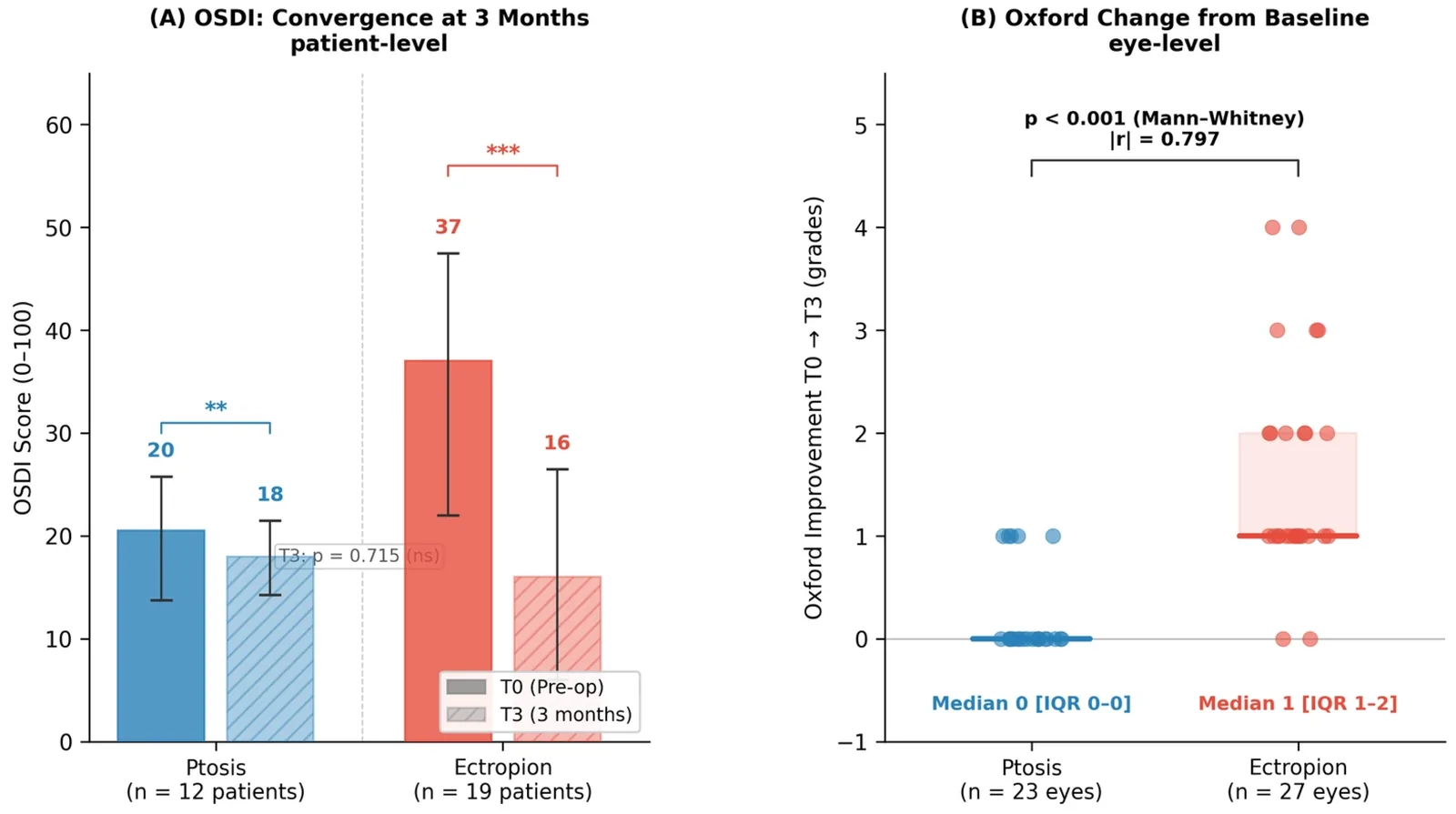
Diagnosis-specific ocular surface outcomes at baseline (T0) and 3 months (T3). (**A**) Patient-level OSDI: baseline did not differ significantly between groups (median 37.0 vs. 20.5, *p* = 0.109); both improved significantly at T3 (within-group Wilcoxon, *p* < 0.001) and converged to comparable values (ptosis median 18 vs. ectropion 16; between-group Mann–Whitney, *p* = 0.715). (**B**) Oxford staining, change from baseline (eye level): median improvement 1 [IQR 1–2] grade after ectropion repair versus 0 [0–0] in ptosis (Mann–Whitney U = 63, *p* < 0.001). Hatched bars represent 3-month values. Error bars = SEM. OSDI, Ocular Surface Disease Index; SEM, standard error of the mean. ** *p* < 0.01; *** *p* < 0.001; ns, not significant.

Oxford staining improvement from baseline to 3 months (analyzed at the eye level) was significantly greater after ectropion repair. When improvement was defined as baseline minus the 3-month score, the median change was 1 grade [IQR 1–2] in the ectropion group versus 0 grades [0–0] in the ptosis group (Mann–Whitney U = 63, *p* < 0.001; |r| = 0.797) (Figure 2); absolute 3-month values (ectropion 0.63 vs. ptosis 0.00) are reported descriptively in Table 3.

**Figure 3:**
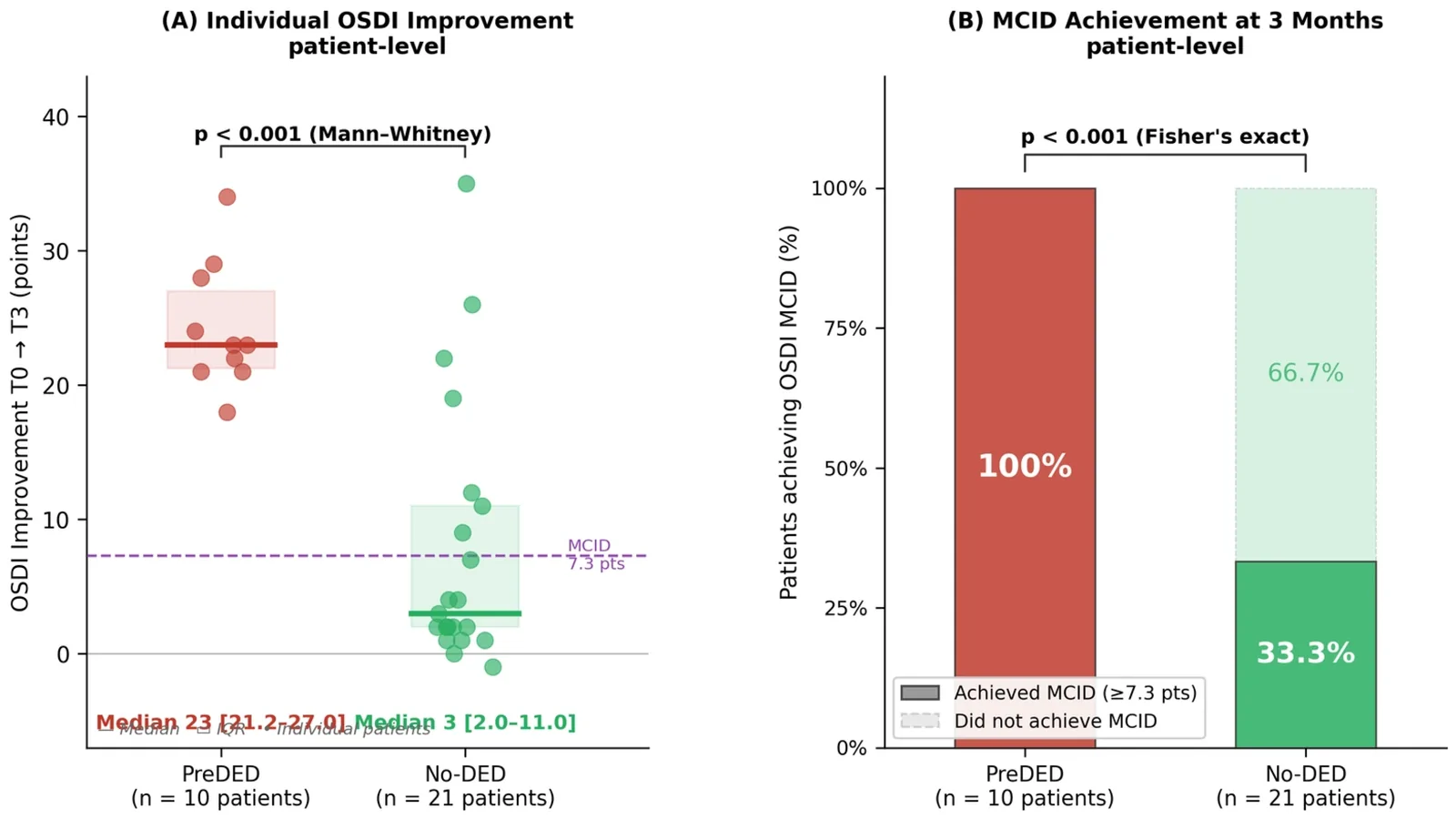
Impact of pre-existing dry eye disease on individual ocular surface recovery. (**A**) Individual OSDI improvement (T0 to T3) in the PreDED and No-DED groups. Each point represents one patient; horizontal bar = median; box = interquartile range. The dashed line indicates the OSDI MCID threshold (7.3 points). Mean ± SEM values are shown below each group. PreDED patients showed substantially greater improvement (Mann–Whitney U test, *p* < 0.001). All PreDED individual data points exceed the MCID threshold. (**B**) Proportion of patients achieving the OSDI MCID (≥7.3 points) at T3: 10/10 PreDED patients (100%) versus 7/21 No-DED patients (33.3%; Fisher’s exact test, *p* < 0.001). PreDED, pre-existing dry eye disease (OSDI ≥ 23 plus ≥ 1 objective sign); MCID, minimal clinically important difference; IQR, interquartile range; OSDI, Ocular Surface Disease Index.

Pre-existing DED impact: Patient-level analysis included 10 patients with pre-existing severe DED and 21 without. Baseline OSDI was significantly higher in the preDED group (median 50.0 [45.25–52.0] vs. 21.0 [11.0–25.0]; *p* < 0.001), and remained higher at 3 months (24.5 [18.25–28.75] vs. 12.0 [6.0–18.0]; *p* = 0.001), although the absolute symptomatic improvement was substantially greater in preDED patients (23.0 [21.25–27.0] vs. 3.0 [2.0–11.0] points; *p* < 0.001; |r| = 0.795). All 10 preDED patients (100%) achieved the OSDI MCID of ≥7.3 points versus 7/21 no-DED patients (33.3%; *p* < 0.001); applying the stringent ≥13.4-point threshold, 10/10 (100%) versus 4/21 (19.0%; *p* < 0.001). Nevertheless, 5/10 preDED patients (50.0%) retained OSDI ≥ 23 at 3 months versus 4/21 no-DED patients (19.0%); this difference did not reach statistical significance (Fisher’s exact test, *p* = 0.105) (Figure 3). Oxford staining (eye level) improved significantly in both groups (preDED: Δ = −1.89; *p* < 0.001; no-DED: Δ = −0.44; *p* < 0.001) (Table 4).

Sensitivity analysis: Quantitative sensitivity analyses for the objective outcomes were done. In GEE models accounting for bilateral-eye clustering, Oxford staining decreased significantly at 3 months in the ptosis reference group (β = −0.217, 95% CI −0.402 to −0.033; *p* = 0.021), with a substantially greater reduction in ectropion (3-month time-by-diagnosis interaction β = −1.375, 95% CI −1.848 to −0.903; *p* < 0.001). Schirmer I showed no significant 3-month effect (β = +0.261 mm, 95% CI −0.097 to 0.619; *p* = 0.153). The one-eye-per-patient analysis (*n* = 32) showed the same overall direction of effect, with Oxford decreasing more in ectropion than ptosis (between-group difference in change 1.267 grades, *p* < 0.001). These analyses showed generally consistent directions and magnitudes of effect, although statistical significance was attenuated in some subgroup analyses because of the reduced sample size.

## 4. Discussion

### 4.1. Main Findings

This retrospective multicenter study provides three clinically relevant observations in patients undergoing corrective eyelid surgery. First, pre-existing severe dry eye disease (preDED) was associated with incomplete symptomatic recovery despite universal achievement of minimal clinically important difference thresholds: all 10 preDED patients achieved OSDI improvement of at least 7.3 points 19, yet 50% retained moderate symptoms (OSDI ≥ 23) at 3 months compared with 19% of patients without baseline DED (*p* = 0.105; inconclusive due to insufficient power).

Second, ectropion repair yielded substantially greater symptomatic improvement than ptosis correction (MCID achievement 79% vs. 17%, *p* = 0.001), yet both groups converged to comparable final symptom scores at 3 months (*p* = 0.715).

Third, ocular surface parameters followed a biphasic course with transient early deterioration followed by sustained improvement beyond the preoperative baseline. The consistent early worsening at 1 week across all objective parameters supports the interpretation that this course reflects genuine mechanical and inflammatory ocular surface disruption rather than measurement variability [28,29]. This early deterioration likely reflects surgical trauma, inflammatory cytokine release, and temporary tear film instability; specific mediators such as IL-6, IL-8, and TNF-α were not measured here but have been implicated in prior studies [30]. In the subset of patients with documented postoperative chemosis, the decline in NI-BUT was notably uniform (Δ = −2.9 ± 0.3 s) compared with that in those without chemosis (Δ = −1.1 ± 1.5 s), consistent with prior evidence that persistent chemosis is strongly associated with symptomatic dry eye after periorbital surgery [31].

While this biphasic pattern aligns with prior reports in cosmetic blepharoplasty showing return to baseline by 3 months [6,28], the present study demonstrates improvement below baseline (OSDI 30.1 → 16.7, Oxford 1.30 → 0.34), suggesting that anatomical restoration of eyelid–globe congruity may restore ocular surface homeostasis beyond simple recovery from surgical trauma.

### 4.2. Pre-Existing Severe DED

The most clinically significant finding is that severe pre-existing DED may be associated with residual symptomatic burden despite clinically meaningful improvement. Despite universal MCID achievement, 50% retained moderate symptoms at 3 months. This has not been previously quantified in reconstructive eyelid surgery using validated MCID-based criteria. While recent evidence suggests pre-existing DED may influence outcomes after levator advancement [18], the magnitude of improvement has not been quantified using MCID thresholds. The present study suggests that even when patients achieve statistically and clinically meaningful improvement (median OSDI reduction 23 points in preDED vs. 3 points in no-DED, *p* < 0.001), a substantial proportion continue to experience symptomatic burden requiring ongoing management.

This paradox may reflect the dual nature of DED in eyelid malposition: a reversible mechanical component and an irreversible chronic inflammatory component. Eyelid malposition increases frictional forces at the lid wiper-ocular surface interface, causing lid wiper epitheliopathy and mechanical tear film disruption. Surgical correction restores normal lid–globe tribology, immediately reducing friction and evaporative loss, explaining the substantial improvement in patients without pre-existing severe disease (median OSDI reduction 3 points) and the dramatic recovery in ectropion patients with baseline exposure (median OSDI reduction 21 points, median Oxford improvement 1 grade). In patients with severe pre-existing DED, residual symptoms may instead reflect a chronic inflammatory component that persists despite anatomical correction. Although not assessed in the present study, mechanisms such as meibomian gland atrophy, goblet cell loss, epithelial metaplasia, and inflammatory mediator upregulation have been proposed to sustain a self-perpetuating ‘vicious circle’ of tear film instability and may plausibly explain this incomplete recovery [15]. The universal MCID achievement in preDED patients (100%) suggests that mechanical correction provides substantial benefit; the 50% residual symptom rate demonstrates that mechanical correction alone cannot reverse chronic inflammatory remodeling.

All 10 preDED patients had severe baseline disease (OSDI ≥ 33), placing them in the highest severity category in which the MCID range extends from 7.3 to 13.4 points [19]. When applying the upper MCID threshold of 13.4 points, 100% still achieved clinically meaningful improvement, yet the residual symptom rate remained unchanged. This consistency indicates that a subset of patients with severe baseline disease experience incomplete symptomatic recovery despite achieving clinically meaningful improvement. Because patients with higher baseline scores have inherently greater room for absolute reduction, the higher MCID achievement in preDED patients should be interpreted as reflecting a greater absolute symptomatic gain rather than a categorically superior treatment response; this is precisely why the persistence of residual symptoms in half of these patients, rather than the MCID rate alone, is emphasized here.

The comparison between preDED and no-DED groups for residual symptoms (50% vs. 19%, *p* = 0.105) did not reach statistical significance due to insufficient power (estimated 35%). A confirmatory study with approximately 45 patients per arm would provide 80% power to detect this difference. Nevertheless, the observed trend has important clinical implications: patients with severe pre-existing DED should be counseled preoperatively that while substantial symptomatic improvement is expected, complete resolution may not occur, and ongoing dry eye management will likely be required postoperatively.

### 4.3. Diagnosis-Specific Course: Ectropion vs. Ptosis

Ptosis and ectropion were pooled only to characterize the overall biphasic temporal course of ocular surface recovery, as both are involutional eyelid malpositions that disrupt ocular surface homeostasis; all substantive between-group comparisons were performed diagnosis-specifically and analyzed separately (Table 3; GEE time-by-diagnosis interaction). To our knowledge, this is among the first studies to directly compare ocular surface recovery after ectropion repair and ptosis correction using multiparametric dry eye assessment [15,16].

At the patient level, ectropion patients demonstrated markedly greater OSDI improvement (median 21 vs. 2 points, *p* = 0.002) and higher MCID achievement rates (79% vs. 17%, *p* = 0.001), yet final symptom scores converged (median 16 vs. 18, *p* = 0.715). Notably, baseline OSDI did not differ significantly between groups at the patient level (*p* = 0.109), indicating that the greater symptomatic improvement after ectropion repair was not simply a function of higher baseline symptom scores. Zloto et al. demonstrated that Müller muscle-conjunctival resection causes persistent dry eye signs at 5-year follow-up compared with upper blepharoplasty alone [15], suggesting that certain surgical approaches may induce irreversible changes in tear film homeostasis. While our cohort underwent levator aponeurosis advancement, the principle remains: surgical correction addresses anatomical malposition but does not eliminate pre-existing tear film pathology. This pattern reflects fundamental differences in pathophysiology and surgical mechanism. The lateral tarsal strip procedure directly restores lid–globe apposition, immediately reducing evaporative tear loss and corneal exposure [17].

Ectropion patients had significantly worse baseline Oxford staining score (2.22 vs. 0.22, *p* < 0.001) and lower tear film stability at Center B (5.59 vs. 9.96 s, *p* < 0.001), providing greater opportunity for measurable improvement. To account for this baseline imbalance, the between-group comparison of Oxford staining was based on change from baseline rather than absolute 3-month values; the greater improvement after ectropion repair remained significant (median change 1 [IQR 1–2] vs. 0 [0–0] grades, *p* < 0.001), and was confirmed by the clustered GEE interaction term (β = −1.375, 95% CI −1.848 to −0.903; *p* < 0.001). The convergence of final symptom scores despite divergent baseline severity argues against regression to the mean as the sole explanation. In contrast, ptosis correction primarily affects blink dynamics and upper lid position, with more subtle effects on tear film distribution. The convergence of final outcomes despite divergent baseline severity suggests both procedures successfully restore ocular surface homeostasis through different mechanisms.

Ptosis patients still demonstrated significant within-group OSDI improvement (*p* = 0.005) and complete resolution of Oxford staining score (baseline 0.22 to final 0.00), indicating objective benefit despite lower MCID achievement rates. The absence of between-group difference in Schirmer I improvement (*p* = 0.131) suggests aqueous tear production is less affected by eyelid malposition type than evaporative parameters, consistent with the predominant role of lid–globe apposition in tear film stability. The lower MCID rate in ptosis patients (17%) reflects their milder baseline symptoms (mean OSDI 23.1): patients with less severe baseline disease require smaller absolute improvements to achieve the same MCID threshold, yet the threshold remains fixed at 7.3 points. This highlights a limitation of applying uniform MCID criteria across heterogeneous baseline severity groups.

### 4.4. Clinical Implications and Risk Stratification

These exploratory findings may inform a risk-stratified approach to perioperative management in reconstructive eyelid surgery and help identify targets for future intervention trials.

Universal prophylactic management. Given the consistent early worsening observed at 1 week across all patients regardless of baseline severity, prophylactic intensive lubrication during the first postoperative month; for example, preservative-free artificial tears and nighttime ointment may be a reasonable empirical strategy, although this was not tested in the present study and requires prospective evaluation.

High-risk patient identification and optimization. Patients with severe pre-existing DED (OSDI greater than or equal to 33 plus objective signs) should be identified preoperatively and counseled that while substantial improvement is expected, complete symptom resolution may not occur. Whether preoperative optimization with anti-inflammatory therapy (topical corticosteroids) or meibomian gland treatment improves outcomes in these patients is an untested hypothesis generated by our observational data and should be evaluated in future randomized trials [31,32]. An algorithmic approach to prevention and management has reduced persistent dry eye symptoms from 10.9% to 2% at 2 months in cosmetic periorbital surgery, though its efficacy in reconstructive eyelid surgery remains to be established in randomized controlled trials [31]. The present study provides preliminary data on the natural course of ocular surface recovery after corrective eyelid surgery and may help inform the design of future interventional trials. Procedure-specific counseling. Ectropion patients can be counseled to expect high rates of clinically meaningful improvement (79% MCID achievement in our cohort), while ptosis patients should be informed that objective improvements in eyelid position and ocular surface integrity are expected, even if symptomatic improvement is more modest. Both groups should be advised that transient worsening during the first postoperative week is normal and that recovery continues through 3 months.

Endpoint selection for future studies. The 3-month timepoint appears to represent stabilization of ocular surface parameters, consistent with prior reports of margin reflex distance stabilization and lagophthalmos resolution by 11 weeks after ptosis surgery [33]. These findings suggest that 3 months may represent a reasonable endpoint for assessing early ocular surface recovery and for planning future interventional studies.

### 4.5. Limitations

This retrospective pilot study has several limitations. The sample size was relatively small (50 eyes of 32 patients), limiting power for subgroup comparisons; notably, the difference in residual symptoms between preDED and no-DED groups did not reach significance (*p* = 0.105) and warrants confirmation in adequately powered cohorts. NI-BUT was measured with two different devices (Keratograph 5M vs. MS-39 OCT), precluding pooled analysis; because each center used a single surgeon and a single device, surgeon, center, and instrument were mutually nested and could not be disentangled, so NI-BUT was reported by center rather than pooled and should be interpreted cautiously. Bilateral eye inclusion was addressed with one-eye-per-patient and GEE analyses; however, OSDI is a patient-reported outcome, so all symptom-based analyses were performed at the patient level. The ptosis and ectropion groups differed in baseline objective severity, particularly Oxford staining; although between-group inference for Oxford was therefore based on change from baseline, residual confounding cannot be excluded, and the greater absolute improvement in ectropion patients may partly reflect greater opportunity for improvement. All preDED patients had severe baseline disease (OSDI ≥ 33), so findings may not generalize to moderate DED (OSDI 23–32); moreover, the OSDI MCID was derived in dry eye populations, and its application to no-DED individuals with low baseline scores should be regarded as exploratory. Follow-up was limited to 3 months, consistent with American Society of Plastic Surgeons guidelines for early outcome assessment 33 and prior evidence of ocular surface stabilization by this timepoint [28], leaving longer-term durability unknown. Finally, the retrospective design limits causal inference and precluded systematic capture of recognized ocular surface modifiers (diabetes mellitus, tear-reducing systemic medications, obstructive sleep apnea) and of standardized anatomical outcomes (MRD1, lid–globe apposition, lagophthalmos, ectropion correction, recurrence). Although no persistent lagophthalmos, wound dehiscence, or malposition recurrence occurred during follow-up, the lack of quantitative anatomical metrics weakens the proposed link between anatomical restoration and ocular surface improvement; future studies should pair the two endpoint types.

## 5. Conclusions

This retrospective multicenter pilot study demonstrates that reconstructive eyelid surgery produces sustained ocular surface improvement, yet preDED was associated with a higher residual symptomatic burden despite universal achievement of clinically meaningful improvement.

Three principal findings inform clinical practice and risk-stratified perioperative management. First, all patients with severe baseline DED (OSDI ≥ 33 plus one sign) achieved the MCID, yet 50% retained moderate symptoms at 3 months. Anatomical correction alone does not eliminate chronic tear film pathology; these patients require preoperative counseling that substantial improvement is expected but complete resolution may not occur. Second, ectropion repair yielded greater symptomatic improvement than ptosis correction (MCID achievement: 79% versus 17%), yet both groups converged to comparable final symptom scores, reflecting different surgical mechanisms restoring ocular surface homeostasis through distinct pathways. Third, objective parameters followed a biphasic trajectory with transient deterioration at one week followed by improvement below preoperative baseline by 3 months, indicating restoration of eyelid–globe congruity beyond simple recovery from surgical trauma.

These pilot findings support a framework for targeted perioperative management based on preoperative risk stratification. Patients with severe pre-existing DED (OSDI ≥ 23 plus objective signs using conservative thresholds: NI-BUT < 5 s, Schirmer I ≤ 7 mm, or Oxford ≥ 2) may represent a subgroup at higher risk of residual symptoms, for whom individualized counseling and potentially intensified postoperative care warrant prospective evaluation. Whether preoperative optimization with anti-inflammatory therapy, meibomian gland treatment, or intensive lubrication improves outcomes in this subgroup warrants investigation in adequately powered randomized controlled trials.

The application of MCID thresholds provides a methodological framework for future oculoplastic trials, enabling precision medicine approaches that distinguish statistically significant changes from clinically meaningful improvements. Future research priorities include prospective trials evaluating preoperative optimization strategies in high-risk patients, comparative effectiveness studies examining surgical techniques that minimize ocular surface disruption, and the development of validated prediction models integrating baseline severity and the malposition type to enable individualized risk stratification and shared decision-making in reconstructive eyelid surgery.

## Figures and Tables

**Table 1:** Baseline characteristics. DED, dry eye disease (OSDI ≥ 23 plus ≥ 1 objective sign). † OSDI is patient-level (*n* = 31); ptosis *n* = 12, ectropion *n* = 19. Objective parameters are eye-level (*n* = 50). NI-BUT analyzed separately by center due to device differences (Keratograph 5M, Center A; MS-39 OCT, Center B). Mann–Whitney U test for continuous variables; Fisher’s exact test for categorical variables. Bold indicates *p* < 0.05.

Characteristic	Overall	Ptosis (*n* = 23)	Ectropion (*n* = 27)	*p*-Value
Eyes, *n*	50	23	27	-
Patients, *n*	31	12	19	-
Age, mean ± SD (range), years	73.0 ± 13.9 (26–92)	72.2 ± 15.1	74.0 ± 13.1	0.66
Female, *n* (%)	14 (44%)	7 (50%)	7 (39%)	0.54
OSDI, median [IQR] (range) †	25.0 [15.5–44.5] (2–54)	20.5 [13.75–25.75] (8–52)	37.0 [22.0–47.5] (2–54)	0.109
Schirmer I (mm), mean ± SD (range)	11.2 ± 3.9 (4–24)	12.0 ± 2.8 (5–15)	10.6 ± 4.6 (4–24)	0.066
Oxford, mean ± SD (range)	1.30 ± 1.43 (0–5)	0.22 ± 0.42 (0–1)	**2.22 ± 1.34 (1–5)**	**<0.001**
NI-BUT (s), Center B, mean ± SD (range)	7.71 ± 2.59 (3.2–12.2)	9.96 ± 0.98 (8.4–12.2)	**5.59 ± 1.66 (3.2–8.1)**	**<0.001**
NI-BUT (s), Center A, mean ± SD (range)	4.99 ± 2.91 (1.47–11.34)	3.26 ± 1.04 (1.47–4.25)	6.15 ± 3.23 (2.68–11.34)	0.070
PreDED, *n* (%) patients/eyes	10 (32%)/18 (36%)	2/4	8/14	-

**Table 2:** Longitudinal outcomes: Friedman test for overall change; Wilcoxon signed-rank post hoc versus baseline with Bonferroni correction (α = 0.017). † *p*-value refers to the T3 versus baseline post-hoc comparison (Wilcoxon signed-rank test). ‡ *p* = 0.033 does not pass Bonferroni threshold. Bold indicates *p* < 0.05.

Outcome	Baseline (T0)	1 Week (T1)	1 Month (T2)	3 Months (T3)	Δ T3 vs. T0	*p*-Value †
OSDI, patient-level, median [IQR] (*n* = 31)	25.0 [15.5–44.5]	34.0 [22.0–50.0]	27.0 [16.5–32.0]	17.0 [10.5–23.5]	−11.0 [2.0–22.5]	**<0.001**
Oxford grade, eye-level, mean ± SD (range) (*n* = 50)	1.30 ± 1.43 (0–5)	1.72 ± 1.57 (0–5)	0.86 ± 0.93 (0–3)	0.34 ± 0.59 (0–2)	−0.96	**<0.001**
Schirmer I (mm), eye-level, mean ± SD (range) (*n* = 50)	11.2 ± 3.9 (4–24)	9.9 ± 3.9 (4–22)	11.3 ± 3.6 (6–20)	11.9 ± 3.3 (6–22)	+0.7	0.033 ‡

**Table 3:** Diagnosis-specific outcomes at 3 months. MCID = minimal clinically important difference OSDI at the patient level; Oxford at the eye level. Between-group inference for Oxford is based on change from baseline (Mann–Whitney U = 63; rank-biserial |r| = 0.797); absolute 3-month values are descriptive only. MCID = OSDI reduction ≥ 7.3 points. Bold indicates *p* < 0.05.

Parameter	Ptosis (*n* = 23)	Ectropion (*n* = 27)	*p*-Value
Patients, *n* (OSDI)	12	19	-
OSDI baseline, median [IQR]	20.5 [13.75–25.75]	37.0 [22.0–47.5]	0.109
OSDI 3 months, median [IQR]	18.0 [14.25–21.5]	16.0 [6.0–26.5]	0.715
OSDI improvement (T0−T3), median [IQR]	2.0 [1.0–3.25]	21.0 [10.0–23.5]	**0.** **002**
MCID ≥ 7.3, *n* (%)	2/12 (16.7%)	15/19 (78.9%)	0.001
Eyes, *n* (Oxford)	23	27	-
Oxford baseline, mean ± SD (range)	0.22 ± 0.42 (0–1)	2.22 ± 1.34 (1–5)	**<** **0.001**
Oxford 3 months, mean ± SD (range)	0.00 ± 0.00 (0–0)	0.63 ± 0.69 (0–2)	-
Oxford improvement (T0−T3), median [IQR]	0 [0–0]	1 [1,2]	**<0.001**

**Table 4:** Impact of pre-existing severe DED. PreDED = pre-existing DED (OSDI ≥ 23 plus ≥ 1 objective sign); all preDED patients had severe baseline disease (OSDI ≥ 33). All values are patient-level. MCID ≥ 13.4 = upper bound of the severity-adjusted range for severe DED. The residual-symptom comparison was inconclusive (estimated power 35%; ~45 patients per arm needed for 80% power). Mann–Whitney U for continuous variables; Fisher’s exact test for categorical variables. Bold indicates *p* < 0.05.

Parameter	PreDED (*n* = 10)	No-DED (*n* = 21)	*p*-Value
OSDI baseline, median [IQR]	50.0 [45.25–52.0]	21.0 [11.0–25.0]	**<0.001**
OSDI 3 months, median [IQR]	24.5 [18.25–28.75]	12.0 [6.0–18.0]	0.001
OSDI improvement, median [IQR]	23.0 [21.25–27.0]	3.0 [2.0–11.0]	**<0.001**
MCID ≥ 7.3 pts, *n* (%)	10/10 (100%)	7/21 (33.3%)	**<0.001**
MCID ≥ 13.4 pts, *n* (%)	10/10 (100%)	4/21 (19.0%)	**<0.001**
Residual symptoms (OSDI ≥ 23), *n* (%)	5/10 (50.0%)	4/21 (19.0%)	0.105

## Data Availability

The data presented in this study are available on request from the corresponding author due to ethical restrictions.

## Abbreviations

The following abbreviations are used in this manuscript:

DED	dry eye disease
GEEs	Generalized Estimating Equations
IQR	interquartile range
LFU	Lacrimal Functional Unit
LTS	lateral tarsal strip
MCID	minimal clinically important difference
NI-BUT	non-invasive tear break-up time
OSDI	Ocular Surface Disease Index
PreDED	pre-existing dry eye disease
r	rank-biserial correlation
SEM	standard error of the mean
TFOS DEWS	Tear Film and Ocular Surface Society Dry Eye Workshop.
